# Unravelling the hidden DNA structural/physical code provides novel insights on promoter location

**DOI:** 10.1093/nar/gkt511

**Published:** 2013-06-12

**Authors:** Elisa Durán, Sarah Djebali, Santi González, Oscar Flores, Josep Maria Mercader, Roderic Guigó, David Torrents, Montserrat Soler-López, Modesto Orozco

**Affiliations:** ^1^Institute for Research in Biomedicine (IRB Barcelona), Barcelona 08028, Spain, ^2^Joint IRB-BSC Research Program on Computational Biology, Barcelona 08028, Spain, ^3^Bioinformatics and Genomics Group, Center for Genomic Regulation and Universitat Pompeu Fabra, Barcelona 08003, Spain, ^4^Barcelona Supercomputing Center, Barcelona 08034, Spain and ^5^Department of Biochemistry and Molecular Biology, University of Barcelona, Barcelona 08028, Spain

## Abstract

Although protein recognition of DNA motifs in promoter regions has been traditionally considered as a critical regulatory element in transcription, the location of promoters, and in particular transcription start sites (TSSs), still remains a challenge. Here we perform a comprehensive analysis of putative core promoter sequences relative to non-annotated predicted TSSs along the human genome, which were defined by distinct DNA physical properties implemented in our ProStar computational algorithm. A representative sampling of predicted regions was subjected to extensive experimental validation and analyses. Interestingly, the vast majority proved to be transcriptionally active despite the lack of specific sequence motifs, indicating that physical signaling is indeed able to detect promoter activity beyond conventional TSS prediction methods. Furthermore, highly active regions displayed typical chromatin features associated to promoters of housekeeping genes. Our results enable to redefine the promoter signatures and analyze the diversity, evolutionary conservation and dynamic regulation of human core promoters at large-scale. Moreover, the present study strongly supports the hypothesis of an ancient regulatory mechanism encoded by the intrinsic physical properties of the DNA that may contribute to the complexity of transcription regulation in the human genome.

## INTRODUCTION

Gene expression in eukaryotes is a complex process regulated by a myriad of molecular mechanisms. The protein recognition of specific DNA sequence motifs located on promoter regions, upstream of transcription start sites (TSSs), has been traditionally considered as the most important regulatory element in transcription (1,2). Nevertheless, after one decade of the postgenomic era, the location of promoters and in particular TSSs still remains surprisingly challenging (3–6). Classical assumptions such as their location 5′ upstream of transcribed regions or their one-to-one correlation with coding genes might actually be oversimplistic. Indeed, sequence signals like transcription factor–binding sites (TFBSs) show little predictive power when applied at the entire genome level. Furthermore, massive annotation projects (7–9) have provided further evidence about the complexity of promoter location and its occurrence in rather unusual genomic regions. These difficulties illustrate that the mechanisms regulating gene expression are not exclusively based on specific interactions between nucleobases located upstream TSSs and regulatory proteins, as they would lead to detectable sequence signals otherwise. Conversely, it seems that the world of DNA regulation is much more intricate and probably involves a myriad of mechanisms, such as the modulation of chromatin structure or epigenetic signatures (10,11).

We and others (12–15) have suggested the existence of a physical code imprinted onto the DNA fiber, which could account for an ancient regulatory mechanism of basal gene expression. Indeed, core promoters and associated TSSs are DNA segments with an intrinsic ability to act as regulatory regions, as they are depleted in nucleosomes and need to bind to a large number of regulatory proteins, which certainly require special physical properties of the DNA fiber. According to this paradigm, we consider that promoters can be defined as regions of unusual physical deformability (13,15,16), which (even in the absence of traditional sequence motifs) might favor either a suitable nucleosome positioning pattern for protein recognition (17) or an effective binding of core promoter-binding proteins and RNA polymerase (12,18). Notwithstanding, genome-wide analysis of the DNA physical properties (13) revealed that ‘promoter-like’ physical signals appear in regions without evidence for real promoters, challenging the existence of a regulatory physical code in DNA, or alternatively, suggesting the presence of many hidden promoter regions in the human genome.

In this manuscript, we have revisited our presumptions about the existence of a physical code involved in gene activity regulation. To this end, we have evaluated de novo promoter predictions arising from the location of regions with unusual physical properties (13). A representative set of suggested (but not annotated) promoters have been analyzed by applying a combination of medium and high-throughput experimental techniques and analyses. Our study demonstrates that a strikingly large number of theoretical predictions, which were considered ‘false positives’ based on the 2007 knowledge, are indeed true promoters. Therefore, we have been able to determine many novel TSSs and core promoters, which were neither detectable by alternative methods nor presenting orthologous sequence signals with known promoters. Most importantly, the present study enables us to redefine promoter signatures and analyze the diversity, evolutionary conservation and dynamic regulation of human core promoters at large-scale. Overall, our findings provide a solid support to the hypothesis that a primitive physical code imprinted in the DNA fiber constitutes a first level of regulation of gene activity.

## MATERIALS AND METHODS

### ProStar promoter predictor

Our ProStar promoter prediction program is able to predict TSSs based on the presence of an unusual profile of physical properties (particularly the DNA helical stiffness) (13), simplifying previous algorithms that use a variety of empirical descriptors with complex translation to mechanistic models (12). As described elsewhere (13,15), stiffness parameters were derived from atomistic molecular dynamics simulations using model oligonucleotides, annotated at the dinucleotide level, and averaged linearly along 500 bp size windows. In short, we performed a large number of molecular dynamics (MD) simulations, computing then the covariance matrices in the helical space at the dinucleotide step [d(X·Y)/d(Z·T)]. Inversion of such matrix yields a 6 × 6 stiffness matrix for each dinucleotide step (13,15). To keep the model as simple as possible, we only considered diagonal elements of the matrix, i.e. the stiffness of DNA in front of pure ‘twist’, ‘roll’, ‘tilt’, ‘rise’, ‘slide’ and ‘shift’ deformations. The average physical property profiles were defined from the analysis of two genomic sequence sets (NCBI36/hg18 human genome release, March 2006), corresponding to known promoters (positive set) or randomly selected sequences (negative set) according to the reference GENCODE annotation (19). ProStar scores a given DNA sequence as ‘promoter’ or as ‘background’ depending on its similarity to the two reference profiles. This is computationally measured by the Mahalanobis distance—a simple statistical metrics widely implemented in clustering and classification analyses (20)—to both promoter and background reference profiles. Using ProStar default parameters, 500 bp long DNA sequences were analyzed at the genome-wide level to locate potential TSSs (13). In this work, putative human core promoters were identified as regions within a window of −1000/+200 bp relative to the ProStar-predicted TSS locations.

### Selection of TSS prediction sets

To be coherent with the ProStar training, we applied our predictor using ENSEMBL (v47) (21) as a reference annotation to select TSSs located at least 1200 bp away from any other annotated TSS. As a result, we obtained a set of putative ‘false positive’, i.e. regions predicted as promoters by their unusual physical properties but which were not experimentally known. We then filtered out those regions that presented >70% of repetitive elements according to the RepeatMasker algorithm (http://www.repeatmasker.org), or that did not allow unique polymerase chain reaction (PCR) primer localization to the human genome assembly by *in silico* PCR BLAT search (http://genome.ucsc.edu). This process yielded 119 genomic regions (1200 bp long) located around 72 putative TSS (note that it was not always technically possible to study promoters located in both directions).

As a negative prediction set, we randomly selected 100 positions, where ProStar suggested no TSS in a 1200 bp window, and for which unique PCR primers could be located. To make the test unbiased, we did not perform any filtering based on the presence of 2006 known promoters in these ProStar negative predictions. Both ProStar-positive and ProStar-negative predicted promoters were subjected to experimental validation.

The positive set was further compared against the latest gene and transcript reference annotations GENCODE (v7) (19) and ENSEMBL (v56) (21) to determine the true positives.

### Luciferase transcription activity assays

We designed hybridization primers suitable for high-GC content regions. The presence of a unique hybridization site was subsequently verified by a BLAT genome alignment (http://genome.ucsc.edu). Primers were ordered in 96-well plates to Sigma-Aldrich. PCR was performed in a 96-well format using AccuPrime GC-rich DNA polymerase (Invitrogen) for the amplification of selected regions. PCR products were analyzed in a 1% agarose gel. Successfully amplified regions were inserted into the promoterless pGL4.21 (luc2P/Puro) vector and ligated through Sfi I restriction sites (Rapid DNA ligation Kit, Roche) that enable directional cloning. *Escherichia coli* competent cells (DH5α, Invitrogen) were transformed with the ligation products. Two independent colonies were selected from each transformant and were verified by sequencing from both the 5′ and 3′ ends. The experimental approach for luciferase activity assays in a high-throughput approach is outlined in Supplementary Figure S1.

Cos-7, Hek293, U2OS, MIA PACA and MDA231 cells were cultured in Dulbeccós Modified Eaglés Media (DMEM) supplemented with 10% of fetal calf serum (FBS). All cultures were grown as a monolayer in a humidified incubator at 37°C in an atmosphere of 5% CO_2_. One day before co-transfection, 2–6 × 10^4^ cells per well were plated in 96-well plates with 100 µl of DMEM without antibiotics. Confluence of 90–95% was achieved by the second day. Transient DNA co-transfections were performed with 0.1 µg of the corresponding pGL4.21/construct plasmid and 0.02 µg of the pGL4.74 (*hRluc/TK*) vector (Promega) using TransFact reagent (Promega) according to the instructions of the manufacturer. DMEM supplemented with 10% FBS was added to the cells 1 h after co-transfection to allow correct growth and protein expression. Dual Luciferase Reporter Assay (Promega) was performed 36 h after co-transfection using a GloMax Multidetection Luminometer (Promega) with dual injector system allowing rapid reagent addition. Light emission was measured 2 s after addition of each of the substrates and integrated over a 10-s interval. The firefly luciferase activity results were normalized with the renilla luciferase activity from the pGL4.74 (hRluc/TK) plasmid to account for differences in transfection efficiency. The previously characterized *SPG4* gene promoter (22) was used to generate positive (S−621/−1) and negative (S−1290/−424) promoter region controls, respectively. Promoter activity was assessed in duplicates and was considered active if it exceeded 3-fold the score of negative control sequences from the normalized threshold value.

After luciferase assays, 80 regions from both the positive and negative promoter sets were further divided into four subsets for further analysis: subset 1 contains 20 high-confidence ProStar sequences with high luciferase activity (PS+L+); subset 2 contains another 20 high-confidence ProStar sequences with low luciferase activity (PS+L−); subset 3, 20 low-scored ProStar sequences with luciferase activity (PS−,L+); and subset 4, 20 low-scored ProStar sequences with no luciferase activity (PS−L−).

### CAGE analysis

To measure transcription initiation in the different promoter subset regions, profiles of cap analysis gene expression (CAGE) 5′-ends were computed. For this purpose, ENCODE stranded CAGE data from polyadenylated cytosolic RNA of seven different cell lines (GM12878, H1-hESC, HUVEC, HeLa-S3, HepG2, K562 and NHEK) and generated in two bio-replicates were used (23–25). For each cell line, CAGE mappings of quality >20 from each of the two bio-replicates were merged, and their distinct 5′-ends extracted (redundancy was removed to avoid considering reverse transcriptase-PCR artifacts as true signal). Every region was subjected to two CAGE analyses, either considering the luciferase-tested 1200 bp region or a 2000 bp equivalent expanded region centered at the TSS. For every cell line, each time a CAGE tag 5′-end was located within and on the same strand as one of the promoter regions, the distance between the CAGE tag 5′-end and the promoter region 5′-end was computed, and the CAGE frequency corresponding to this distance (further normalized using percentage distance bins) was increased.

### RNA-seq analysis

To measure transcription activity in the different promoter subset regions, profiles of RNA-seq 5′-ends were computed. For this purpose, ENCODE CSHL stranded paired-end RNA-seq data from polyadenylated cytosolic RNA of seven different cell lines (GM12878, H1-hESC, HUVEC, HeLa-S3, HepG2, K562 and NHEK) were used (25). For each cell line, all the mappings of the second bio-replicate were considered, and their distinct most 5′-ends extracted. Every subset region was expanded to a final length of 2000 bp centered at the TSS, similarly to the CAGE analyzed sequences. For every cell line, each time an RNA-seq mapping 5′-end was located within and on the same strand as one of the promoter regions, the distance between the RNA-seq 5′-end and the promoter region 5′-end was computed, and the RNA-seq frequency corresponding to this distance (further normalized using percent distance bins) was increased.

### Chromatin structure and epigenetic signals

The chromatin structure was inferred from DNase I hypersensitivity sites as reported in ENCODE through the UCSC Table Browser data retrieval tool (26). From these data, we calculated the average of Dnase I hypersensitiviy clusters within 1200 bp regions of the different CAGE analyzed subsets, considering a positive cluster when overlapped with the reference promoter elements. We also explored potential epigenetic markers in the suggested promoter regions by looking at the occurrence of histone variants H3KMe1, H3K27Ac and H3K4Me3 in seven different cell lines (GM, H1, HSMM, HUVEC, K562, NHEK and NHLF). For each CAGE analyzed subset of 1200 bp regions, we calculated the number of regions that overcome a certain average alignment density (intensity signal) in any of the different cell types. Using a threshold of 10-fold, 92% of PS+ sequences contained stronger signals compared with the 37% of PS−. Increasing the threshold, up to 50, produced a reduction of the total number of regions, but increased the difference between PS+ and PS− in the same direction.

### TFBS enrichment evaluation

We investigated if different subsets, including the PS+ predictions (17 909 in total), the experimentally tested PS+ predictions (119 sequences) and PS− predictions (100 sequences) or luciferase positive (49 sequences) and negative (23 sequences) regions, were enriched within the 1200 bp in any of the currently annotated 885 TFBSs. To this end, we systematically compared them with a full list of transcripts described in the BioMart database (http://www.biomart.org) (76 905 transcripts) as a background control. To determine the significant enrichment, we used a Fisher’s exact test and represented the magnitude of enrichment as odds ratios, which is the ratio of enrichment for a given TFBS. The corrected significant *P*-value after applying a Bonferroni’s correction for all tests was 0.05/885 = 5.65 × 10^−^^5^. The analyses were performed using the R statistical environment (http://www.r-project.org).

### Core region DNA element conservation and sequence-based signals

The conservation of the four different CAGE analyzed 1200 bp regions was evaluated by the comparison with available vertebrate genomes using the University of California, Santa Cruz (UCSC) Table Browser data retrieval tool (26). The level of conservation for each particular fragment was calculated according to Vertebrate Basewise Conservation by PhyloP as the average of conservation of all nucleotides comprising the region. TFBS conservation was determined from the comparison of boxes among human, mouse and rat according to the UCSC TFBS conservation track using matrices obtained from TRANSFAC database (27). In addition, we used Regulatory region Local Alignment (ReLA) algorithm (28), a footprinting-based program for the detection of conserved clusters of TFBSs, to determine whether the regions predicted by ProStar would also be detectable as sequence-based only promoter signals.

## RESULTS

### Selection of the TSS prediction sets based on DNA physical properties

If physical signals were indeed significant in regulatory regions, as we presume, we would expect a high proportion of ProStar predictions to be promoters, despite the lack of experimental annotation. As described elsewhere (13), promoter sequences provide a distinct profile for six descriptors of the DNA stiffness in front of ‘twist’, ‘roll’, ‘tilt’, ‘rise’, ‘slide’ and ‘shift’ deformations, particularly in regions spanning −250/+900 bp relative to TSSs (that is, covering core and proximal promoter distances).

**Figure 1.**
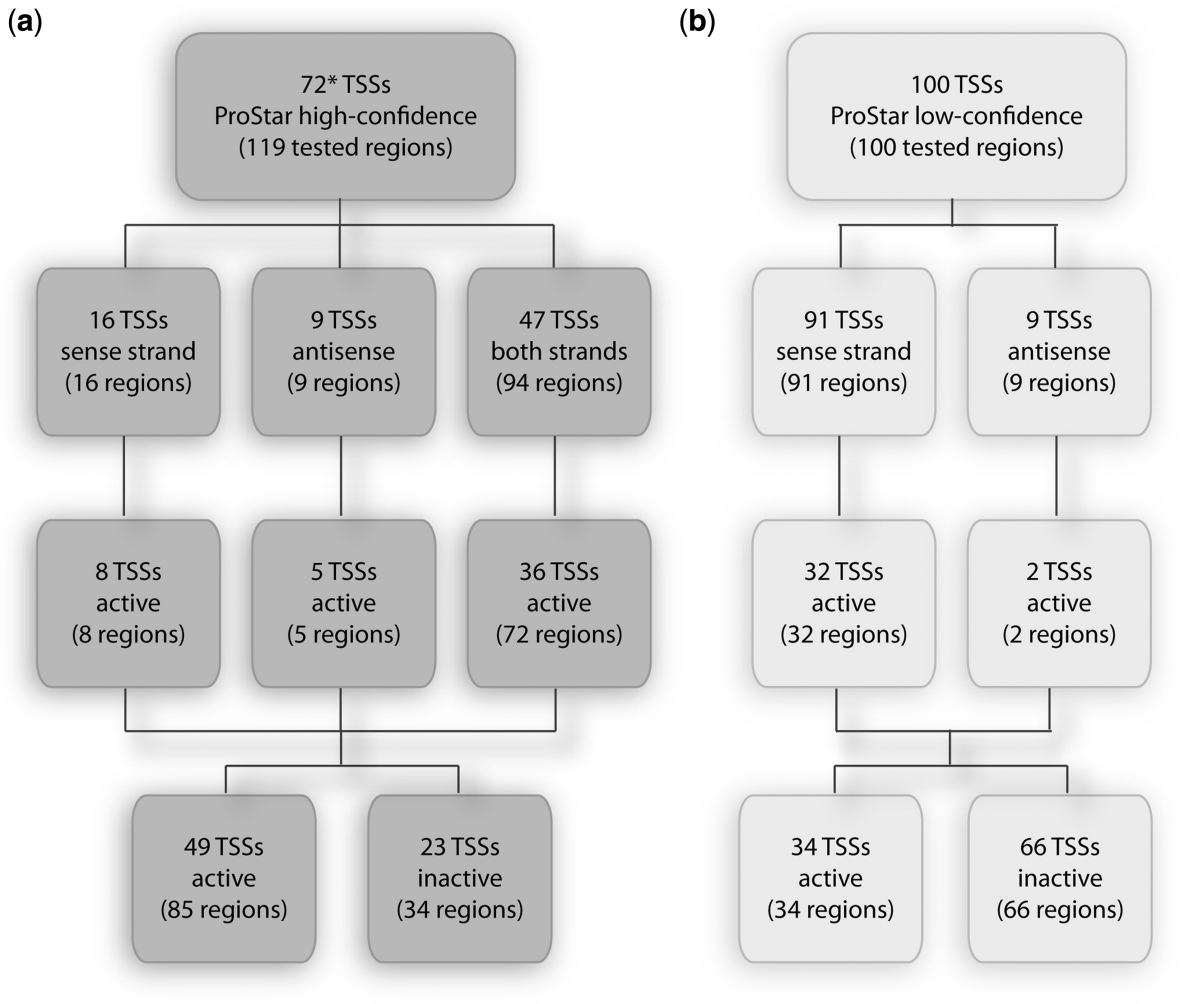
Identification of functional promoters. Summary scheme of TSS selection for both positive (**a**) and negative (**b**) ProStar sets, classified based on luciferase activity (3-fold) and directionality of tested regions: sense, antisense or both sense strands. (Asterisk) 24 out of 72 TSSs were annotated on recent transcriptome reference annotations (21) based on the 2009 genome release (GRChR37/hg19), i.e. true positives.

Therefore, to validate our hypothesis, we first defined a TSS prediction set for experimental screening from the ProStar genome-wide calculations. To better validate the prediction power, we used the original ProStar outcome based on the 2006 release of the human genome (13), without retraining the software with more recent releases of genome data. We selected regions with unusual physical properties suggested to be promoters albeit they were not annotated in reference databases, i.e. ProStar ‘false positives’ (PS+, see ‘Materials and Methods’ section). Furthermore, even though the algorithm recognizes the directionality of transcription, predictions might also account for bidirectional regulatory elements. Thereby, we selected 72 high-scored putative TSSs that allowed unique PCR primer hybridization to the human genome assembly on the sense-strand (16 TSSs), antisense (9 TSSs) or in both senses (47 × 2 TSSs), yielding 119 different putative promoter regions in total (Figure 1a, Supplementary Table S1). We additionally defined a negative set consisting of 100 sequences corresponding to nonsignaled promoter regions by ProStar (PS−, 91 on direct-sense and 9 anti-sense due to PCR constraints) (Figure 1b, Supplementary Table S1).

Comparison of the physical deformability properties between both sets revealed the distinct underlying features that had allowed ProStar to recognize the positive TSS set as putative promoter regions, as described above (15). When we further compared our positive set against the latest transcript reference annotations GENCODE (v7) (19) and ENSEMBL (v56) (21), 24 predicted TSSs appeared to be functional (i.e. they are certainly true positives), giving an unexpected support to the quality of our physical de novo predictions (Supplementary Table S1). Yet, up to 48 ProStar predicted regions are not proximate (<1.2 kb) to any 2012-annotated TSS. Intriguingly, the attempt of validating ProStar predicted regions using methods based on interspecies sequence conservation, such as ReLA (28), yielded a low success rate (9%), providing further evidences that ProStar locates putative promoters in genomic regions where phylogenetic footprinting finds no signal.

### Identification of functional promoters

We evaluated the ability of the selected putative regions to activate transcription in mammalian cells by using luciferase reporter gene expression assays (Supplementary Figure S1). By applying a threshold of at least 3-fold higher activity than the control vehicle, 85 putative promoters regions were scored as functional, with a validation rate of 71.4%, while only 34% of the analyzed regions in the negative set displayed activity (Figure 1; Supplementary Table S1). From those 85 positively active sequences, 8 correspond to sense strand, 5 to antisense and 72 to both directions (i.e. putative bidirectional promoters or alternative regulatory elements), accounting for 49 distinct TSSs. Interestingly, a significantly large number of the suggested promoters (37.8%) displayed high activity (10-fold above the vehicle). Furthermore, almost 98% of active promoters in one cell line also displayed activity in three additional cell lines, indicating that the identified regions would mainly generate transcripts involved in housekeeping activities, rather than in tissue-specific processes. Taken together, these findings suggest that physical properties would signal promoters of the loosely regulated ‘housekeeping’ genes, whereas highly specific sequence signals would be required for the activation of development or tissue-specific genes.

### CAGE and RNA-Seq analyses in support of predicted TSSs

Luciferase measurements showed that the vast majority of ProStar TSS-derived regions function as promoters when coupled to a reporter gene and transfected to mammalian cells (Figure 1; Supplementary Table S1). Nevertheless, we should also consider that the resulting activity could be an artifact for some regions, as the activity measurements were based on plasmid-inserted regions rather than on their native structure like in bulk chromatin. Alternatively, the activity might result from the absence of methylation or other posttranslational modifications of a true cellular environment, which can modify the DNA physical properties and ultimately lead to a transcription repression *in vivo* (29–31).

Consequently, we complemented our first validation with a CAGE (7,32,33) to examine the transcription start activity of the experimentally tested 1200 bp regions in living cells (25). We selected 80 regions showing different levels of luciferase activity and classified them into four representative categories. Subset 1 contains 20 ProStar high-scored sequences with high luciferase activity (PS+L+); subset 2 contains another 20 ProStar high-scored sequences with low luciferase activity (PS+L−); subset 3, 20 low-scored ProStar sequences with luciferase activity (PS−L+); and subset 4, 20 low-scored ProStar sequences with no luciferase activity (PS−L−) (Supplementary Table S1).

**Figure 2.**
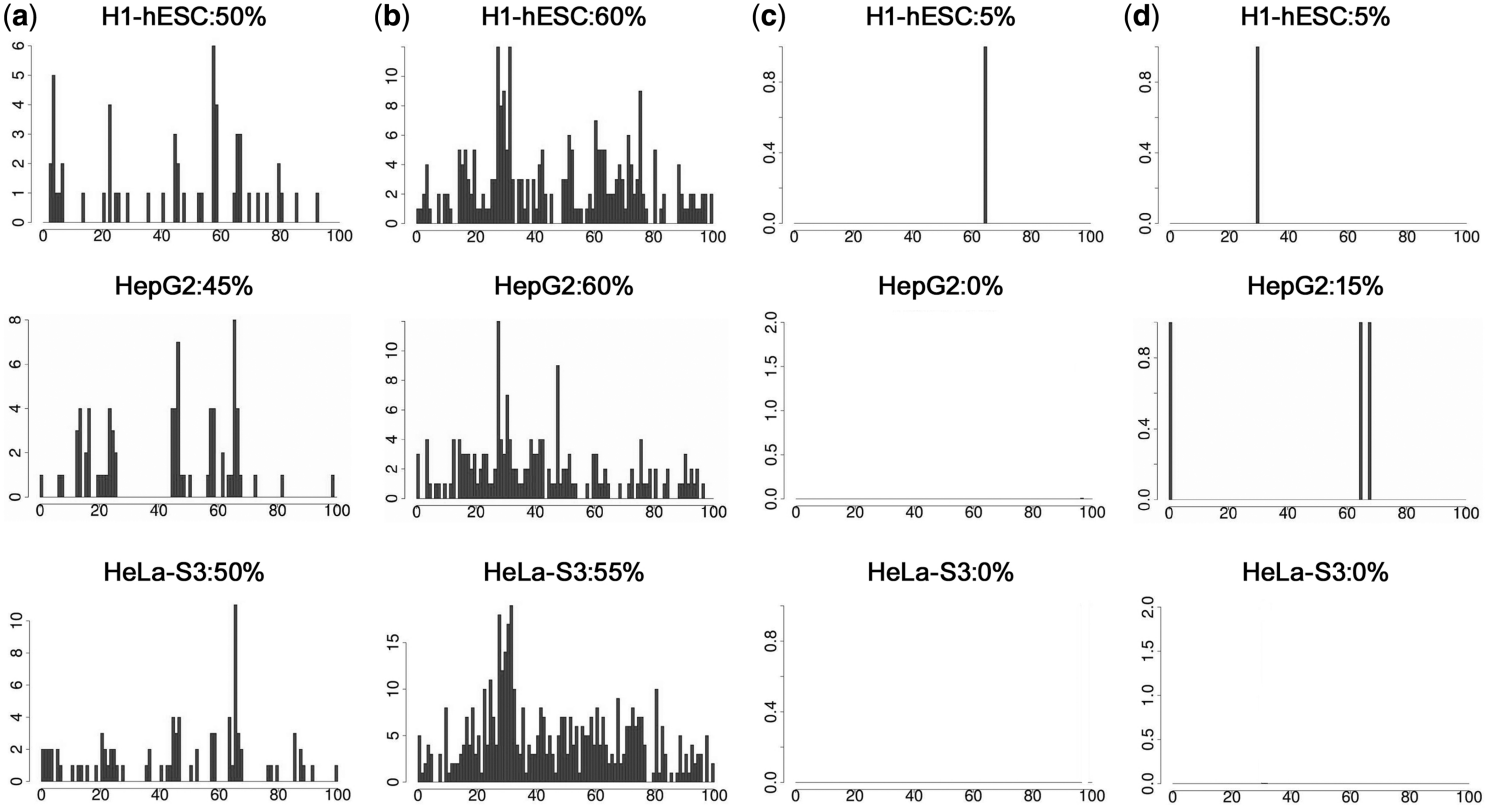
Orthogonal support of predicted TSSs: CAGE analysis. Distribution of distinct 5′-ends of CAGE tags from several representative CAGE experiments in H1-hESC, HepG2 and HeLa-S3 cell types based on cytosolic polyA+ transcripts. For every distinct most 5′-end of CAGE tag detected within and on the same strand as a particular promoter region, we increased the CAGE frequency of the percent distance bin corresponding to the distance between the CAGE tag 5′-end and the promoter region 5′-end. As the predicted promoter regions were 1200 bp long, each % distance bin includes 12 bp, and thereby the TSS is expected to be located on the 84th distance bin (i.e. at 1000 bp from the region 5′-end). (**a**) PS+L+ subset 1. For most of the cell types, the major peak appears around the 63th bin (i.e. 750 bp), closely matching with the prediction (**b**) PS+L− subset 2. We observe undefined peaks around the 30th–50th bins (350–600 bp). On the other hand, the number of CAGE tags is significantly higher than for subset 1 (**c**) PS−L+ for subset 3, (**d**) PS−L− for subset 4. ProStar negative PS− subsets clearly show an almost inexistent CAGE signal.

The results summarized in Figure 2 show that regions from subsets 1 (PS+L+) and 2 (PS+L−) were dramatically enriched for CAGE tags that could be confidently mapped to single positions (Figure 2a and b), as compared with the ProStar negative subsets 3 (PS−L+) and 4 (PS−L−) (Figure 2c and d). Subset 1 displayed the highest proportion of sequences with CAGE tagged 5′-ends around 750 bp, indicating that those regions contained reliable TSS marks (Figure 2a, around 60th distance bin). CAGE tags were detected in most of the human cell type experiments, but a particular enrichment was found for polyadenylated (polyA+) transcripts, suggesting that active regions might correspond to promoter elements regulating protein-coding genes.

Interestingly, subset 3 (PS−L+) regions contain few cage tags, although they showed some activity in luciferase expression assays (Figure 2c). We could simply assume that this subset contains luciferase-false positives. However, it has been reported that the structure of promoters on different chromosomes varies and these variations might not be well covered by whole-genome promoter prediction algorithms (6). Thus, we cannot rule out the possibility that promoters located in anomalous positions, and hence harboring a divergent pattern of physical properties, could have been overlooked by ProStar (13,15). If these regulatory elements turn out to be under tight regulation in bulk chromatin (which would explain why no CAGE tags are detected), they could well show transcriptional activity in luciferase assays, which ignore activation or inhibition signals imprinted in the native chromatin structure.

Even more intriguingly, subset 2 regions (PS+L−) did show clear CAGE enrichment although they did not provide a luciferase response (Figure 2b). These discrepancies could simply result from luciferase-false negatives. However, the strength and the profile of CAGE signals (Figure 2b) indicated that other factors could also account for the low luciferase/high CAGE response. Comparison of the CAGE profiles indicated that subset 1 peaks are located at the expected TSSs (i.e. around 60th bin; Figure 2a), while subset 2 peaks are upstreamly displaced from the original prediction (around 30th bin; Figure 2b). These findings suggest that, under certain conditions, physical properties are able to signal promoter regions although the prediction of the TSS location can be upstreamly displaced from the true site. In this scenario, CAGE experiments would still detect transcript 5′ in the −1000/+200 bp analyzed genomic window. On the other hand, this displacement would have led us to amplify truncated promoter constructs undetectable by the conservative luciferase test we initially applied in our experimental workflow (Supplementary Figure S1).

**Figure 3.**
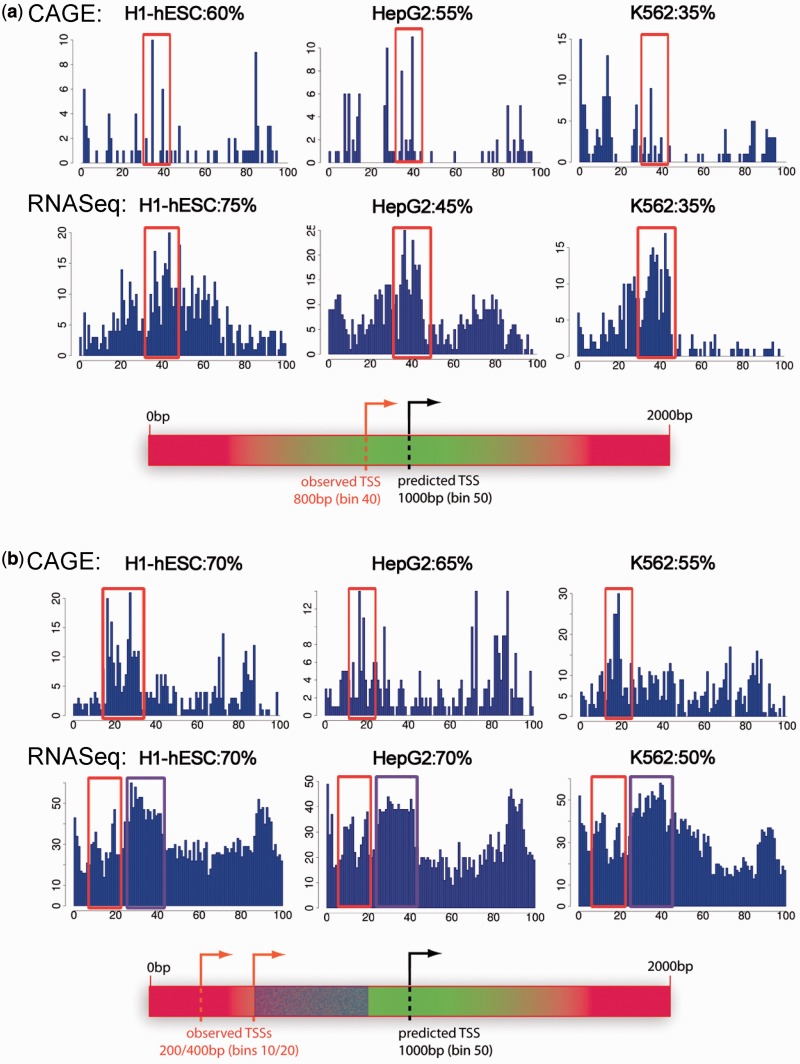
Orthogonal support of predicted TSSs: CAGE vs RNA-seq analyses. Distribution of 5′-ends of CAGE/RNA-seq tags from representative CAGE/RNA-seq experiments in H1-hESC, HepG2 and K562 cell types based on cytosolic polyA+ transcripts. The profiles were constructed similarly to the 1200 bp CAGE analysis. However, as the predicted promoter regions were now 2000 bp long, each % distance bin includes 20 bp and hence the predicted TSS should be located on the 50th distance bin (i.e. 1000 bp), as it is indicated in the promoter region schematic representations below the profiles (**a**) PS+L+ subset 1. The observed TSSs extrapolated from CAGE and RNA-seq profiles appear around the 40th bin (i.e. 800 bp, highlighted with orange frames), and closely match the predictions (**b**) PS+L− subset 2. We observe two sharp RNA-seq peaks around the 10th–20th bins (200–400 bp) that match with CAGE peaks around the 20th bin (highlighted with orange frames). Furthermore, a broad peak is observed right after the observed TSSs, indicating that it may correspond to a transcription active region (i.e. an exon, highlighted in purple) but not necessarily a transcription start region.

To validate this hypothesis, we carried out RNA-sequencing (RNA-seq) analysis to survey the transcription profiles of the selected regions and to identify putative exons near the suggested TSSs. We performed the analysis of 2000 bp regions centered on the predicted TSSs, using RNA-seq data of subcellular-fractionated RNAs from the ENCODE Consortium (Supplementary Figure S2, see ‘Materials and Methods’ section for details) (9,25). Interestingly, the profiles of subset 1 presented a sharp RNA-seq peak at 800 bp, which coincided with the CAGE major peak around 750 bp (Figure 3a, around 40th bin, orange frames). Furthermore, this TSS putative peak was corroborated with a downstream peak corresponding to an exon (50–80th bins, i.e. from 1000 to 1400 bp) in most of the cell lines. Conversely, subset 2 profiles showed two sharp RNA-seq peaks at 200 and 400 bp, respectively, which matched CAGE major peaks around 360 bp (Figure 3b, 10–20th bins, highlighted with orange frames). Moreover, a downstream broad peak likely corresponding to an exon (Figure 3b, 20–40th bins, i.e. from 400 to 800 bp, highlighted with purple frames) could further confirm the TSS displaced positions at ∼700–500 bp upstream relative to predictions.

**Figure 4.**
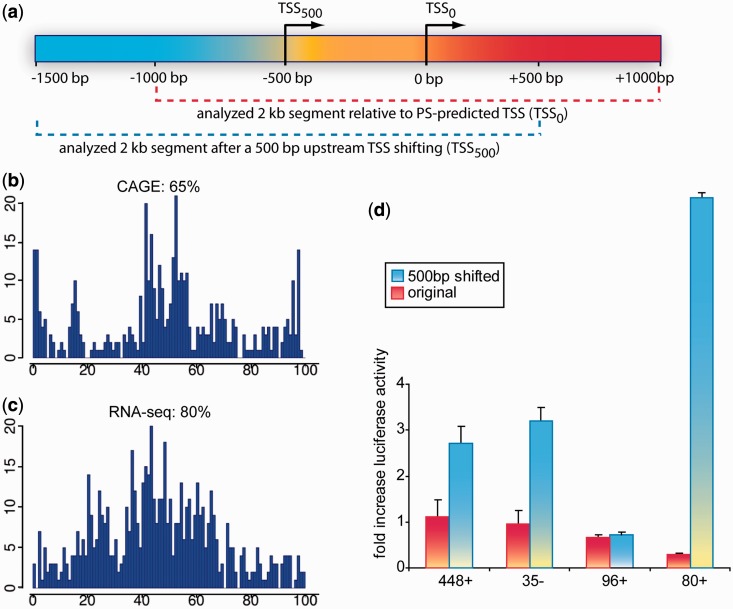
Evaluation of PS+L− sequences on centering the TSS 500 bp upstream from the prediction. (**a**) Subset 2–shifted regions were reconstructed by first re-locating the TSS 500 bp upstream from the relative prediction in the human genome, and subsequently selecting the flanking ±1000 bp upstream and downstream regions, respectively (**b**) Distribution of CAGE tags in H1-hESC cells for the 2000 bp regions centered in relocated TSSs (**c**) RNA-seq analysis profiles of the same regions. X-axes show % distance bins, each one including 20 bp. Y-axes display the number of detected tags. Here we observe a major peak from both analyses around the 50th bin (1000 bp), indicating that it may correspond to a transcription start region (**d**) We confirmed the transcription ability of those regions by additional luciferase assays in four representative PS+L− sequences (three in sense strand and one in anti-sense), showing a significant higher activity (green bars) as compared with the original predictions (red).

We further interrogated this potential TSS displacement in the prediction by analyzing new genomic fragments but now centered on the observed CAGE peaks. To this end, we picked up regions from subset 2 and placed the TSS 500 bp upstream to the original ProStar TSS prediction, as indicated by the CAGE/RNA-seq profiles (Figure 4a). As expected, CAGE profiles exhibited a major peak around 800–900 bp, resembling subset 1 sequences (Figure 4b, around 45th bin). Similarly, RNA-seq profiles also presented a single peak at the expected position (Figure 4c, around 50th bin, 1000 bp). We then re-amplified four of these genomic regions by PCR, spanning 2000 bp but centered at the newly located TSS, as similarly done with previous subsets (Figure 4a; see Supplementary Figure S1 for method details). Interestingly, luciferase assays measured a 4-fold higher activity on average than the original sequences (Figure 4d), providing further evidence that subset 2 segments (PS+L−) do contain true TSSs.

### Core promoter activity landscape

We subsequently analyzed the four subsets of ProStar predictions to seek for correlations between structural or epigenetic motifs and the promoter activity status. To this end, we used data repositories publicly available from the ENCODE Consortium (9) (See ‘Materials and Methods’ section for details).

**Figure 5.**
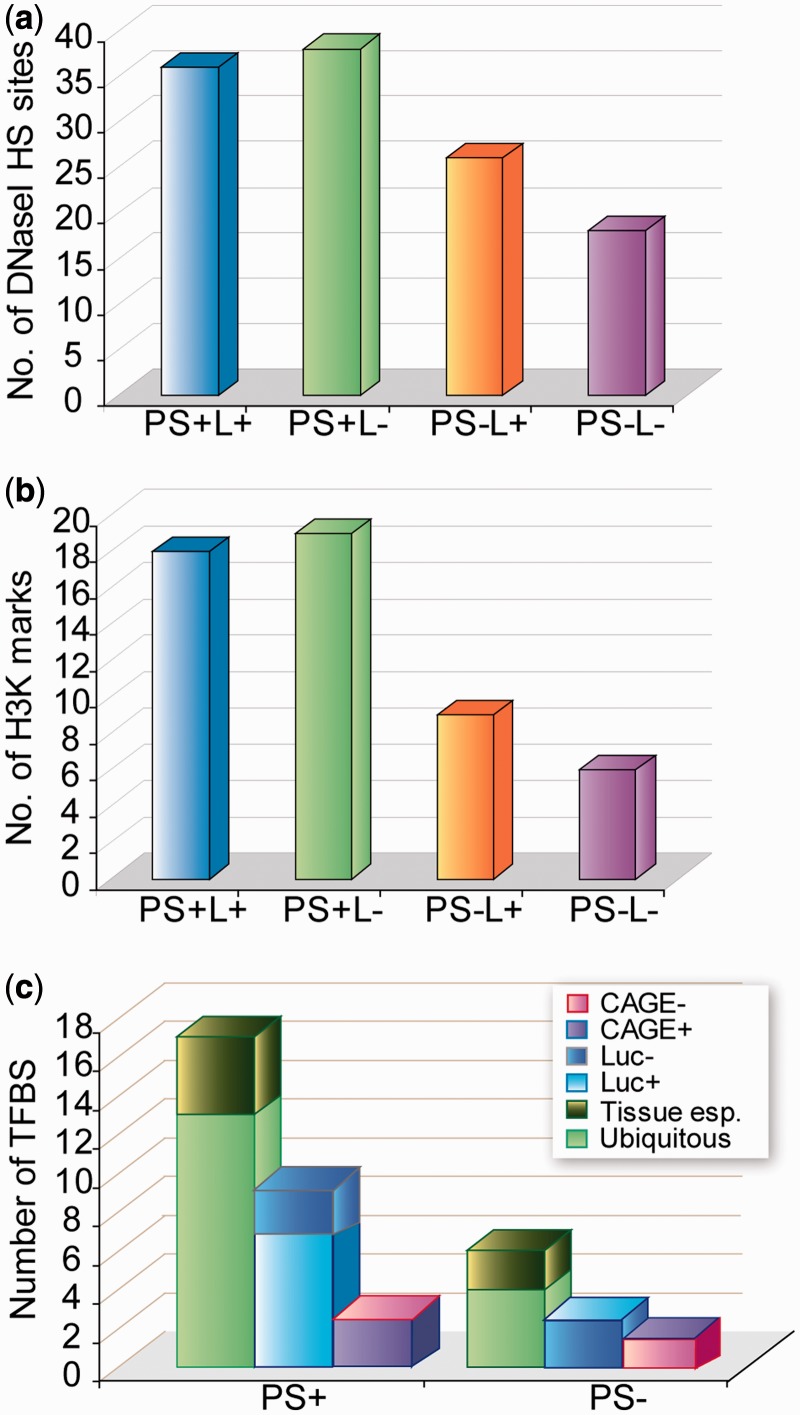
Putative promoter activity landscape. (**a**) Average DHS enrichment within 1200 bp regions of the different CAGE analyzed subsets in a large collection of cell types available in ENCODE (**b**) Average plots of detected Histone 3 variants correlating with transcriptional activity: H3K4Me1, often observed near regulatory elements; H3K27Ac, occurring near promoters; H3K4Me3, near active regulatory elements (**c**) TFBS enrichment evaluation of PS+ and PS− predictions according to gene expression (i.e. ubiquitous vs tissue-specific; left column in the respective PS+ and PS− groups). Further evaluation of TFBS enrichment in the experimentally tested PS+ and PS− sequence sets according to luciferase transcription activity (Luc+ or Luc−, middle bars) and CAGE mapping analysis (CAGE+ or CAGE−, right bars).

We first analyzed chromatin accessibility to DNase I degradation profiles, as DNase I hypersensitive sites (DHS) are expected to correlate with loosely packed regions in bulk chromatin and hence with gene transcriptional activity (34–36). Analysis of ENCODE data (Figure 5a) highlighted a similar DHS density for ProStar positive subsets (PS+L+ and PS+L−), which turned out to be much larger than the observed density for the negative subsets (PS−L+ and PS−L−). These observations indicate that ProStar-predicted regions are indeed open and thereby associated with transcriptionally active chromatin. Of note, those predictions cannot be simply explained on the basis of sequence-dependent rules such as the presence of CpG islands, as the CG content provides a disperse prediction signal and leads to large number of false positives (13). It should also be noted that ProStar is able to detect promoters located at a large distance to any annotated CpG island (37), as this is the case for 20% of the positive predictions analyzed by CAGE (Supplementary Table S2).

We also evaluated the occurrence of histone modifications correlated with epigenetic modulation of gene transcription, in particular H3K4Me1, H3K27Ac and H3K4Me3, which are specifically prevalent in regulatory regions (38). The results shown in Figure 5b revealed that these histone marks were actually more overrepresented in ProStar positive regions (PS+L+ and PS+L−) than in ProStar negative regions (PS−L+ and PS−L−), providing accumulating evidence about the attainable implication of ProStar regions in the regulation of gene activity.

Furthermore, as PS+ regions are located on regulatory elements, we quested for potential associations to specific functions by a TFBS enrichment evaluation, using the Transfac database (27). To this end, we examined diverse region subsets, including all PS high-scored predictions (PS+, 17909 sequences), the experimentally tested regions (containing 119 PS+ predictions and 100 PS− predictions, respectively) and the CAGE-analyzed sets (subsets 1 and 2 on the one hand, and subsets 3 and 4 on the other hand). The enrichment for a given TFBS was considered to be significant when *P* < 5.65 × 10^−^^5^ (Supplementary Table S3). Again, PS− regions showed little enrichment, whereas 17 human TFBSs were found to be overrepresented in at least one of the PS+ groups, the larger part being annotated as ubiquitous (Figure 5c, left column in PS+ and PS− groups, respectively) and mostly related to vital cellular functions (Supplementary Table S3). Interestingly, TFBSs overrepresented in the regions capable of driving luciferase transcription were also enriched in PS+ predictions (Figure 5c, middle bars, Luc+) and CAGE tagged sequences (Figure 5c, right bars, CAGE+). In addition, the identified TFBSs presented high binding affinity to GC-rich sequences, representing truly active TFBSs and thereby supporting our hypothesis that ProStar accurately predicts promoters of housekeeping genes.

**Figure 6.**
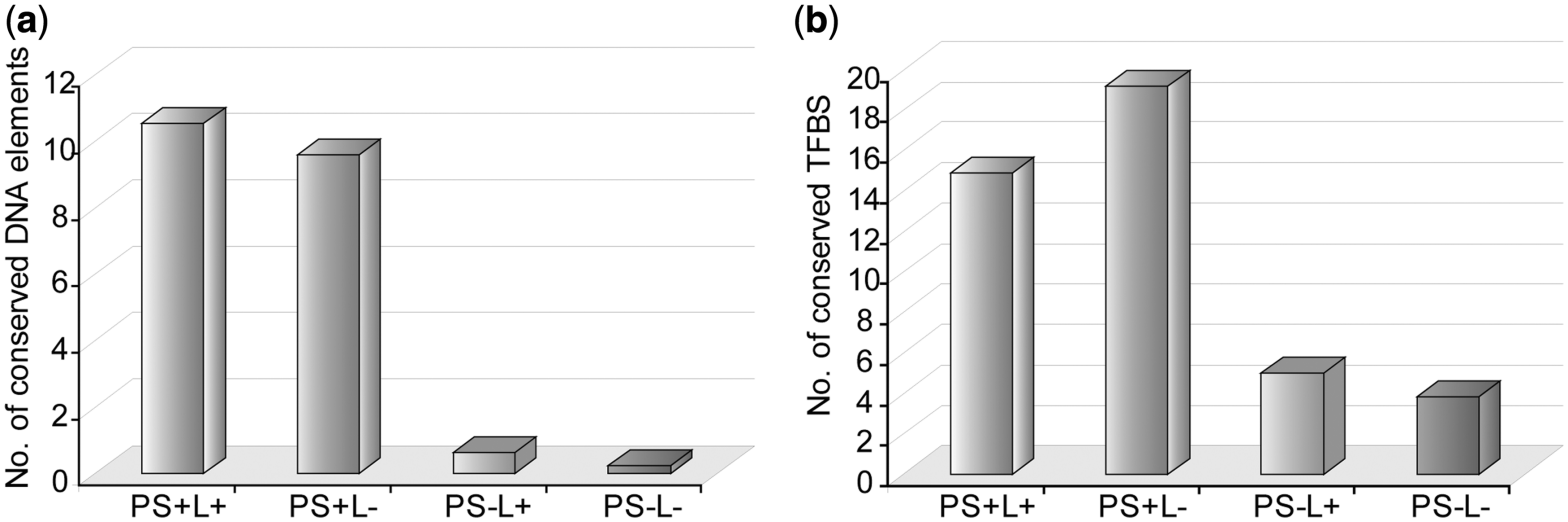
Putative promoter interspecies conservation. (**a**) Average plots of identified DNA elements conserved among human, mouse and rat using the ‘Vetebrate PhyloP’ algorithm within 1200 bp regions of the different CAGE analyzed subsets (**b**) Similarly, plot histograms showing the average number of identified TFBSs that are conserved across species.

Lastly, although the vast majority of the PS+ regions were not detectable using phylogenetic footprinting-based methods (as we previously discussed), we further investigated the conservation of ProStar regions across species, as biologically relevant sequences should display some level of sequence conservation. As expected, PS+ regions were enriched for conserved DNA elements (Figure 6a), particularly for TFBSs (Figure 6b), as compared with PS− regions.

## DISCUSSION

A comprehensive analysis of ProStar predicted TSSs has enabled us to identify novel functional core promoters in the human genome exclusively detected by their differential physical deformability pattern and not simply by sequence-based signals such as the CG content alone. A large percentage of ProStar seemingly ‘false positives’, i.e. regions with unusual physical properties but not associated to any annotated promoter, are indeed transcriptionally active. In particular, highly active regions containing a differential physical pattern display typical chromatin features of housekeeping gene promoters involved in cell survival and maintenance, as proven by an overwhelming amount of direct (luciferase assays, CAGE or RNA-seq mapping) and indirect evidence (profile analysis such as DNaseI sensitivity, epigenetic markers, TFBS enrichment or DNA element conservation). Interestingly, physical signaling also appears to be able to detect promoter activity even in cases where the TSS is located 500 bp upstream of the prediction. Whether this displacement is indicative of a particular feature of genes with closely related alternative TSSs, as indicated by massive CAGE and RNA-seq mappings (Figures 3b and 4), will nevertheless require further investigation. Taken together, these observations reinforce the evidence that high-confidence ProStar predicted regions, sharing a defined pattern of physical features, truly behave like physiologically active TSSs.

We have also observed that most of the active core regions signaled by physical properties do not exhibit directionality in transcript initiation, indicating that physical properties might signal zones where the binding of regulatory proteins and the deformation of DNA are less intricate, as we had previously suggested (39–42). Yet, this signaling might not be sufficient to determine the correct sense of transcription. Intriguingly, more than half of all human promoters are bidirectional, and hence directionality of promoter activity may be regulated to some degree in a cell type–specific manner (43).

On the whole, our study provides insights into the role of DNA physical properties in ascertaining an ancestral coarse regulatory mechanism. Thereby, regions with high chance of undergoing spontaneous transcription would be recognized by protein effectors and favor nucleosome depletion aside from the purely sequence-based signals encoded as H-bond patterns in the DNA major and minor grooves. In fact, recent genome-wide nucleosome mapping analyses from our group have revealed that housekeeping genes display unique nucleosome architectures, with large nucleosome refractory regions upstream the TSS (unpublished data). In general then, the interplay between DNA physical properties and regulatory regions could be rationalized in terms of nucleosome positioning (16), favoring the presence of sequences with unique deformation properties in promoter regions, although this might not be the only underlying mechanism, and this would probably vary from gene to gene.

Yet, the physical code type of mechanism could have been evolutionary deactivated in specific genes where fine regulation is required, but seems to be still active in many other cases, where such a stringent regulation is not essential. This convoluted regulatory signaling present in complex organisms could partially explain the failure of traditional promoter location methods to identify a significant number of TSSs, implying the presence of many hidden promoter regions in the human genome.

## SUPPLEMENTARY DATA

Supplementary Data are available at NAR Online: Supplementary Tables 1–3 and Supplementary Figures 1 and 2.

## FUNDING

Spanish Ministry of Science and Innovation [BIO2012-32868 and Consolider E-Science Project]; Instituto de Salud Carlos III (Instituto Nacional de Bioinformática); European Research Council (ERC) Advanced Grant; Fundación Marcelino Botín. M.O. is an Institució Catalana de Recerca i Estudis Avançats (ICREA) Academia Researcher. Funding for open access charge: Fundación Marcelino Botín.

*Conflict of interest statement.* None declared.

## Supplementary Material

Supplementary Data
