# Supplementary material for: Unravelling the hidden DNA structural/physical code provides novel insights on promoter location
Source: Nucleic Acids Res. 2013 Jun 12;41(15):7220–30. doi: 10.1093/nar/gkt511 (PMC3753636; doi:10.1093/nar/gkt511)
Supplement: Supplementary Data [file supp_41_15_7220__index.html]

Unravelling the hidden DNA structural/physical code provides novel insights on promoter location — Unravelling the hidden DNA structural/physical code provides novel insights on promoter location — Supplementary Data 

# Unravelling the hidden DNA structural/physical code provides novel insights on promoter location

## Supplementary Data

files

**Files in this Data Supplement:**

- Supplementary Data - zip file
