# Supplementary material for: Unravelling the hidden DNA structural/physical code provides novel insights on promoter location
Source: Nucleic Acids Res. 2013 Jun 12;41(15):7220–30. doi: 10.1093/nar/gkt511 (PMC3753636; doi:10.1093/nar/gkt511)
Supplement: Supplementary Data [file supp_gkt511_nar-01209-n-2013-File008.zip › NAR-01209-2013 Suppl files/Duran_et_al_Supplementary_Figures_S1-S2.pdf]

Unraveling the hidden DNA structural/physical code provides novel insights on promoter location

Elisa Durán, Sarah Djebali, Santi González, Oscar Flores, Josep Maria Mercader, Roderic Guigó, David Torrents, Montserrat Soler-López and Modesto Orozco

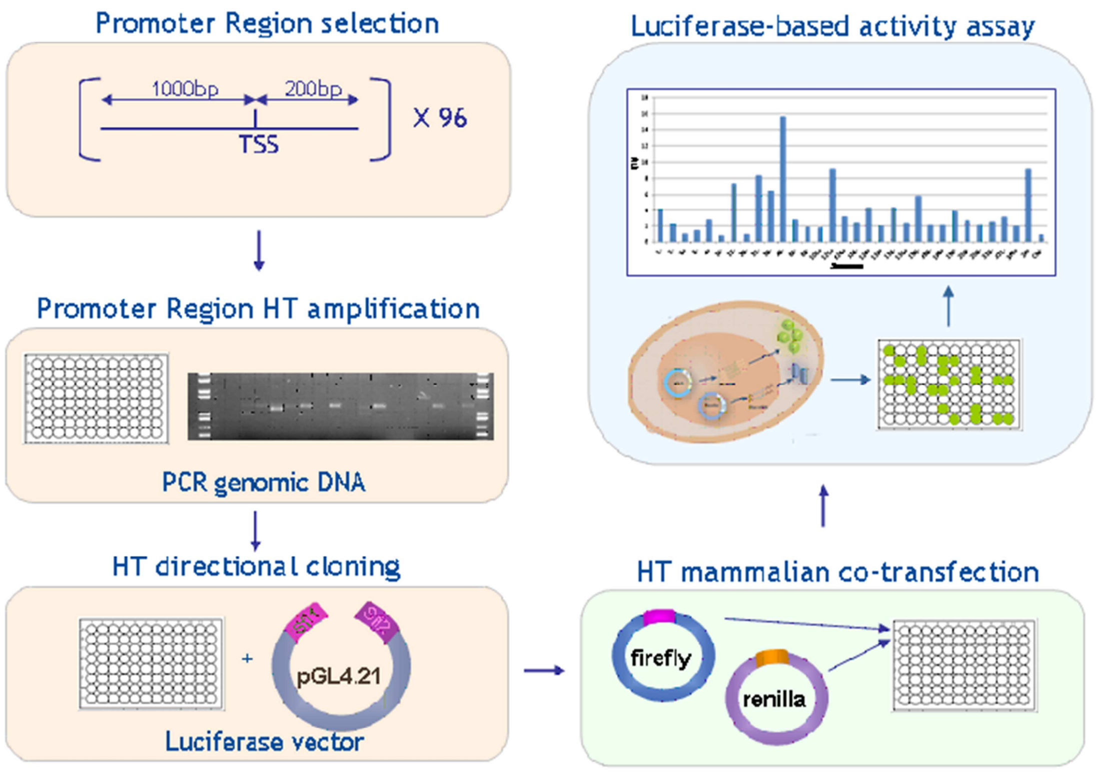

Supplementary Figure 1. Experimental approach for luciferase activity assays in a high-throughput approach. Five major steps: (1) selection and amplification or putative promoter regions (2) high-throughput (HT) PCR amplification of selected fragments from human genomic DNA (3) HT fragment cloning into the promoter-less vector upstream of the firefly luciferase encoding gene (4) transient co-transfection in mammalian cells with a constitutive luciferase expressing vector (renilla) for value normalization (5) firefly luciferase measurements to evaluate the potential promoter activity of selected fragments.

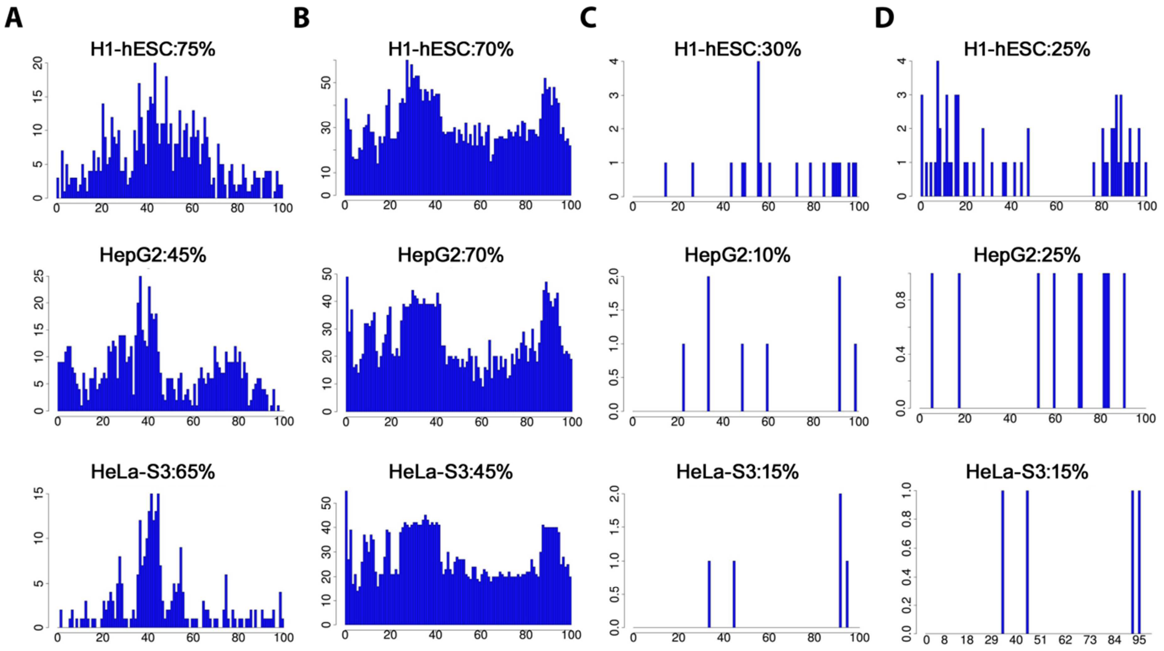

Supplementary Figure S2. Orthogonal support of predicted TSSs: RNA-seq analysis. Distribution of distinct sequenced tags from several representative RNA-seq experiments in H1-hESC, HepG2 and HeLa-S3 cell types based on cytosolic polyA+ transcripts. For every distinct most 5'-end of RNA-seq tag detected within and on the same strand as a particular promoter region, we increased the RNA-seq frequency of the percent distance bin corresponding to the distance between the RNA-seq tag 5'-end and the promoter region 5'-end. As the predicted promoter regions were 2000 bp long, each % distance bin includes 20 bp and thereby the TSS is expected to be located on the 50th distance bin (i.e. at 1000 bp from the region 5'-end). (a) PS+L+ subset 1. For most of the cell types, the major peak appears around the 40th bin (i.e. 800 bp), closely matching with the prediction (b) PS+L- subset 2. We observe undefined peaks around the 10th-20th bins (200-400 bp). On the other hand, the number of RNA-seq tags is significantly higher than for subset 1 (c) PS-L+ subset 3 (d) PS-L- subset 4. ProStar negative PS- subsets clearly show an almost inexistent RNA-seq signal.
