# Supplementary material for: Unravelling the hidden DNA structural/physical code provides novel insights on promoter location
Source: Nucleic Acids Res. 2013 Jun 12;41(15):7220–30. doi: 10.1093/nar/gkt511 (PMC3753636; doi:10.1093/nar/gkt511)
Supplement: Supplementary Data [file supp_gkt511_nar-01209-n-2013-File008.zip › NAR-01209-2013 Suppl files/Duran_et_al_Supplementary_Table_2.pdf]

Unraveling the hidden DNA structural/physical code provides novel insights on promoter location

Elisa Durán, Sarah Djebali, Santi González, Oscar Flores, Josep Maria Mercader, Roderic Guigó, David Torrents, Montserrat Soler-López and Modesto Orozco

Supplementary Table 2. Correlation of physical deformability patterns with the CG content. ProStar positive predictions analyzed by CAGE that are located at ≥ 3500 bp distance to any annotated CpG island, based on UCSC Genome Browser. The relative distance to the closest annotated CpG islands is indicated as base pairs (bp). (*) The negative symbol refers to the strand orientation.

Subset	Chromosome	Predicted TSS location (GRCh37/hg19)	Tested region range 1,2kb (GRCh37/hg19)	Distance (bp) to the closest annotated CGI*
G1 (PS+/L+)	7	61.820.762	61820562..61821762	693.753
	1	40.335.364	40334364..40335564	13.577
	4	62.068.131	62067931..62069131	314.882
	5	138.897.705	138897505..138898705	42.879
G2 (PS+/L-)	4	147.576.411	147576211..147577411	-14.510
	14	60.981.788	60981588..60982788	-3.608
	18	76.752.776	76752576..76753776	-11.532
	19	920.551	920351..921551	4.237
